# Persistent Symptoms among Frontline Health Workers Post-Acute COVID-19 Infection

**DOI:** 10.3390/ijerph19105933

**Published:** 2022-05-13

**Authors:** Constance Wose Kinge, Susan Hanekom, Alison Lupton-Smith, Francis Akpan, Eula Mothibi, Thapelo Maotoe, Floyd Lebatie, Pappie Majuba, Ian Sanne, Charles Chasela

**Affiliations:** 1 Implementation Science Unit, Right to Care, On the Lake, 1006 Lenchen North Avenue, Centurion 0046, South Africa; charles.chasela@righttocare.org; 2 Department of Epidemiology and Biostatistics, Faculty of Health Sciences, School of Public Health, University of the Witwatersrand, Johannesburg 2000, South Africa; 3 Physiotherapy Division, Faculty of Medicine and Health Sciences, Stellenbosch University, Fancie van Zijl Drive, Tygerberg, Cape Town 8000, South Africa; sdh@sun.ac.za (S.H.); aluptonsmith@sun.ac.za (A.L.-S.); 4 Right to Care, On the Lake, 1006 Lenchen North Avenue, Centurion 0046, South Africa; francis.akpan@equiphealth.org (F.A.); eula.mothibi@righttocare.org (E.M.); thapelo.maotoe@equiphealth.org (T.M.); floyd.lebatie@righttocare.org (F.L.); pappie.majuba@righttocare.org (P.M.); ian.sanne@righttocare.org (I.S.); 5 Clinical HIV Research Unit, Faculty of Health Sciences, School of Clinical Medicine, University of the Witwatersrand, Johannesburg 2000, South Africa

**Keywords:** COVID-19 virus infection, post-acute COVID-19 syndrome, persistent COVID-19 symptoms, healthcare workers

## Abstract

Growing evidence shows that a significant number of patients with COVID-19 experience persistent symptoms, also known as long COVID-19. We sought to identify persistent symptoms of COVID-19 in frontline workers at Right to Care South Africa, who are past the acute phase of illness, using a cross-sectional survey. We analysed data from 207 eligible COVID-19 positive frontline workers who participated in a two-month post-COVID-19 online self-administered survey. The survey response rate was 30%; of the 62 respondents with a median age of 33.5 years (IQR= 30–44 years), 47 (76%) were females. The majority (*n* = 55; 88.7%) self-isolated and 7 (11.3%) were admitted to hospital at the time of diagnosis. The most common comorbid condition reported was hypertension, particularly among workers aged 45–55 years. The most reported persistent symptoms were characterised by fatigue, anxiety, difficulty sleeping, chest pain, muscle pain, and brain fog. Long COVID-19 is a serious phenomenon, of which much is still unknown, including its causes, how common it is especially in non-hospitalised healthcare workers, and how to treat it. Given the rise in COVID-19 cases, the prevalence of long COVID-19 is likely to be substantial; thus, the need for rehabilitation programs targeted at each persistent COVID-19 symptom is critical.

## 1. Introduction

Since the outbreak of coronavirus disease 2019 (COVID-19) and the unprecedented and rapid spread of the disease resulting in high morbidity and mortality [1], scientific evidence suggests that 10% of patients with COVID-19 experience symptoms beyond 3–4 weeks [2], especially in hospitalised patients [3]. In a study by Huang et al. [4], more than 75% of patients who were previously hospitalised with COVID-19 had at least one lingering symptom six months later. Some suggested residual effects of SARS-CoV-2 infection reported so far include but are not limited to fatigue, shortness of breath, joint pain, chest pain, cognitive disturbances, and a drop in the quality of life [3,4]. Growing evidence shows that a significant number of patients with COVID-19 experience prolonged/persistent symptoms, also known as long COVID-19. Reports of long COVID-19 are rising, especially with the continued rise in cases. Though some insights into long COVID-19 have been achieved in the past year in hospitalised patients suffering acute COVID-19, much is still unknown with respect to its prevalence in non-hospitalised frontline healthcare workers who remain at the forefront of the pandemic, including the duration of the symptoms and the management of the symptoms.

South Africa reported its first case of COVID-19 on 5 March 2020 [5]; since then, there has been a rise in infection, especially among frontline health workers. The novel coronavirus SARS-CoV-2 poses an even greater risk to frontline health workers than any other infections previously reported [6]. By 6 May 2020, South Africa reported 511 cases in healthcare workers (HCWs) (7% of the national total), with nurses accounting for 53% of total HCW cases [7]. With each wave of infection, HCWs have continued to experience a high number of infections as well as mortality; and for those who have passed the acute phase of infection, they are required to return to work after the minimum amount of time possible.

Frontline healthcare workers at Right to Care South Africa, a non-profit organisation operating across seven provinces providing health services, have not been spared by the advent of COVID-19 and would likely not be spared post-acute sequelae of SARS-CoV-2 infection. In this study, we sought to identify and determine the prevalence of persistent/long COVID-19 symptoms among frontline workers at Right to Care (RTC) South Africa who are past the acute phase of illness. This was to better understand how widespread, severe, and for how long have these persistent symptoms lasted.

## 2. Materials and Methods

### 2.1. Design and Participants

An anonymous cross-sectional survey was launched on an online platform from 15 February to 15 April 2021 in South Africa. All RTC staff were eligible for inclusion if (1) they were working either on a permanent or short-term basis across seven provinces (Gauteng, Free State, Eastern Cape, Northern, North-West, and Mpumalanga) in South Africa where RTC operates and (2) had tested positive for COVID-19. Staff were excluded if they (1) had inconclusive, negative, pending, and missing COVID-19 results, and (2) invalid, missing phone and/or emails. Participants were directed to the survey site by clicking the survey link, which by so doing provided informed consent. No IP address was collected. Approval to conduct the survey was provided by the Right to Care COVID-19 Business Continuity Committee and the Health Research Ethics Committee (HREC) of Stellenbosch University (SU), South Africa (HEA-2020-19255).

### 2.2. Research Setting

The study was conducted among Right to Care (RTC) South Africa staff. RTC is a non-governmental organisation (NGO) working in response to public healthcare emergencies including HIV and AIDS, tuberculosis (TB), as well as emerging outbreaks such as Ebola and COVID-19. COVID-19 testing for the organisation is carried out at private AMPATH and Lancet laboratories, as well as at the National Health Laboratory Services (NHLS), a government institution using real-time reverse transcription-polymerase chain reaction (RT-PCR) performed on either nasopharyngeal swabs (NPS) or oropharyngeal swabs (OPS as per guidelines [8]. The test results are sent to the designated RTC Medical Officer and captured on the RTC COVID-19 database.

### 2.3. Survey Instrument

The survey questionnaire was developed by the Right to Care (RTC) Implementation Science (IS) team in partnership with the COVID-19 Business Continuity Committee, and the Stellenbosch University research team. It consisted of 67 items covering the following seven aspects: (1) demographics (age, sex, and ethnicity), (2) general health status (smoking, alcohol, and recreational drug use), (3) comorbidities, (4) health status at the time of COVID-19 diagnosis, (5) health status post-COVID-19, (6) current health status post-COVID-19, and (7) physical activity. Participants were asked to rate how bothersome the post-COVID-19 symptoms were, using a 10-point end labelled adopted response scale (least bothersome to most bothersome). Similarly, for the severity of current post-COVID-19 symptoms, a 10-point end labelled slider scale was adopted for the response scale (not noticeable to unbearable). Activity vital signs were used to determine participants’ activity levels [9].

### 2.4. Data Collection

The online self-administered questionnaire—survey link—was distributed to eligible employees (to reach as many staff as possible) via email, SMS, and WhatsApp, with information about the objectives as well as the conditions of participation and data confidentiality provided. These different platforms were used to increase the response rate. A weekly reminder was sent out via email as well as SMS and WhatsApp for the duration of the survey period, which was active for a two-month period. All participation was voluntary, and responses were anonymous. The collected data were password-protected and only the SU/RTC Implementation Science (IS) researchers had the access code to log in for using the data for analysis.

### 2.5. Statistical Analysis

Data management and analysis were conducted using Stata/SE Version 15.1. Descriptive information including the demographics of respondents, health status, underlying medical conditions, post-acute COVID-19 as well as persistent (long) COVID-19 symptoms, and physical activity levels were calculated. Long COVID-19 was defined as the presence of symptoms for four weeks and beyond in 2020 [6]. In recent times, the definition has been revised to mean the presence of symptoms lasting “usually three months on from the onset of the COVID-19 with symptoms that last for at least two months and cannot be explained by an alternative diagnosis” [10]. Differences between categorical variables were determined using Chi-square or Fisher’s exact tests (for sparse data). A *t*-test was used to compare the statistical difference in means of days and time spent on physical activity before COVID-19 diagnosis and after recovery. The Mann–Whitney U-test was used to compare health mean scores disaggregated by sex. Statistical significance was considered when *p*-values were <0.05.

## 3. Results

### 3.1. Demographics

**Figure 1:**
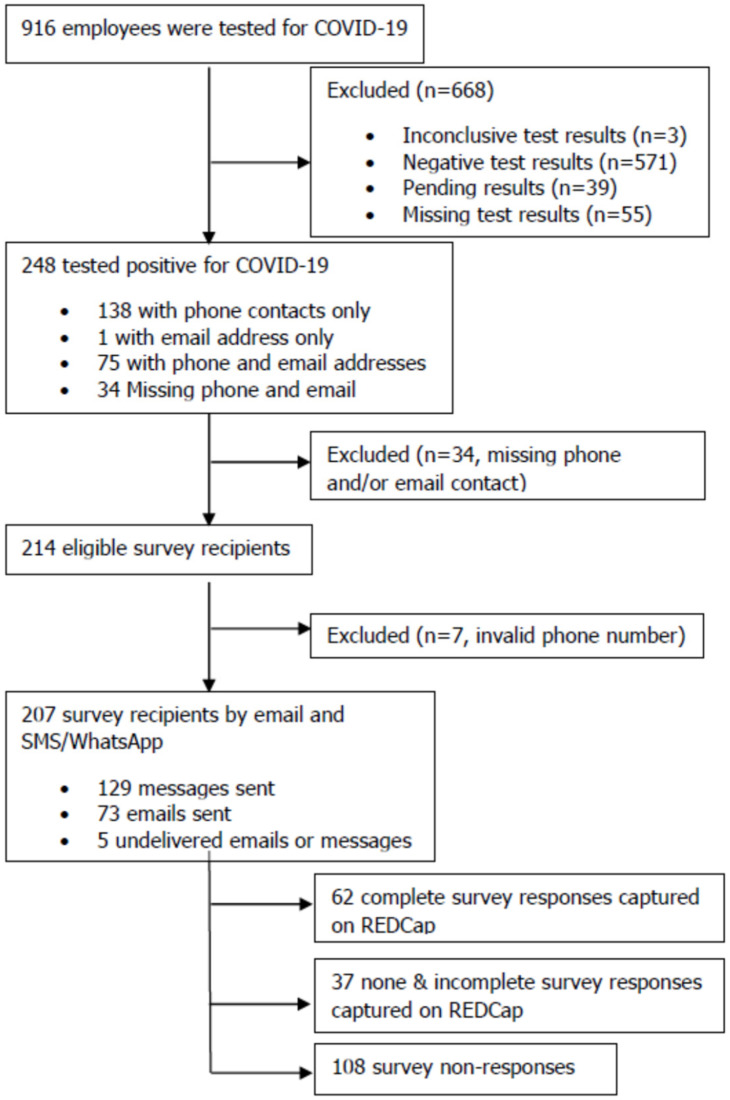
Participant flow chart. Data for frontline workers who had tested positive for SARS-CoV-2 by RT-PCR between 5 January 2020 and 7 February 2021 were extracted from the RTC database. Exclusion and inclusion criteria were applied resulting in 207 eligible participants.

A total of 916 employees were tested for COVID-19 between 5 January 2020 and 7 February 2021 and captured on the RTC COVID-19 database. Of these, 248 (27%) tested positive, 207 were eligible to take part in the survey, 99 (47.8%) were invited via 129 SMS/WhatsApp messages and 73 emails to participate, and only 62 (giving a response rate of 30%) consented and completed the survey as shown (Figure 1). Of the 248 staff who tested positive for COVID-19, approximately 38% (*n* = 95) and 17% (*n* = 42) were community/field health workers and medical/clinical personnel, respectively as shown (Supplementary Table S1). COVID-19 person under investigation (PUI)/suspect was the main reason for testing, accounting for 61% (*n* = 151) of positive cases notably in the Free State (33%; *n* = 81), Mpumalanga (25%; *n* = 61) and Western Cape (28%; *n* = 70) provinces. The highest-hit district municipalities were Thabo Mofutsanyane (29%; *n* = 73), Ehlanzeni (23%; *n* = 58), and Overberg (10%; *n* = 25). Staff who tested positive were significantly different (*p* < 0.05) from others (negative, pending, inclusive, and missing test results) across cadres, provinces, districts, and reasons for testing (Supplementary Table S1).

The median age of the respondents (*n* = 62) was 33.5 years (interquartile range (IQR) = 30–44 years), the majority, 31 (50%), were between the ages of 23–33 years, and 47 (~76%) were females (Table 1). African (87%; *n* = 54) was the most common ethnicity, followed by coloured (~10%; *n* = 6). The majority (~86%; *n* = 53) had never smoked and 6.4% (*n* = 4) were current smokers. Of the four current smokers, each had been smoking <10 cigarettes per day for the past 3, 5, 10, and 14 years, respectively.

**Figure 2:**
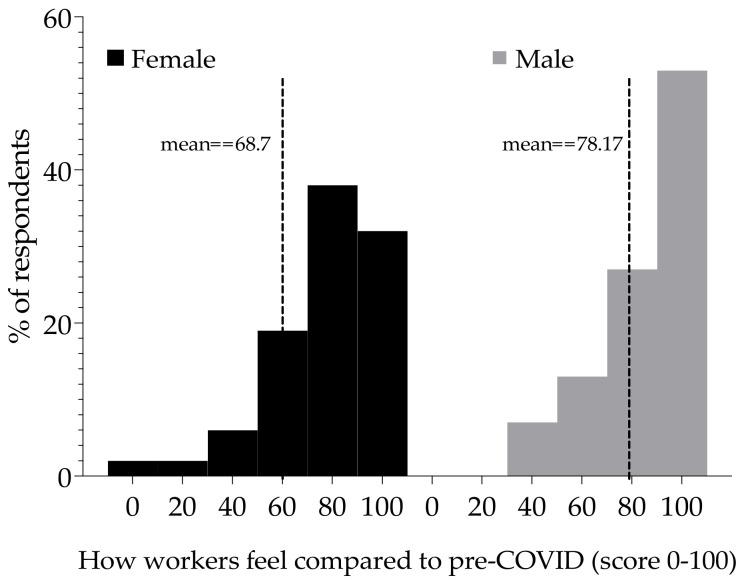
Health scores for respondents post-acute COVID-19 symptoms. Distribution of health scores (0–100) for female respondents (*n* = 47) and male (*n* = 15). Zero (0) to mean worst health and 100 best health. The separated (grouped) bars show the percentage of respondents that reported the scores. The vertical dashed lines indicate the mean scores for each distribution.

On the day of the survey, which was a period between one week to one year after initial diagnosis, respondents were asked, “we would like to know how good or bad your health is TODAY” (Figure 2). Female respondents reported a mean score of 68.7, while males reported a mean score of 78.1 (*p* > 0.05; Mann–Whitney U-test).

### 3.2. Co-Morbidities

**Figure 3:**
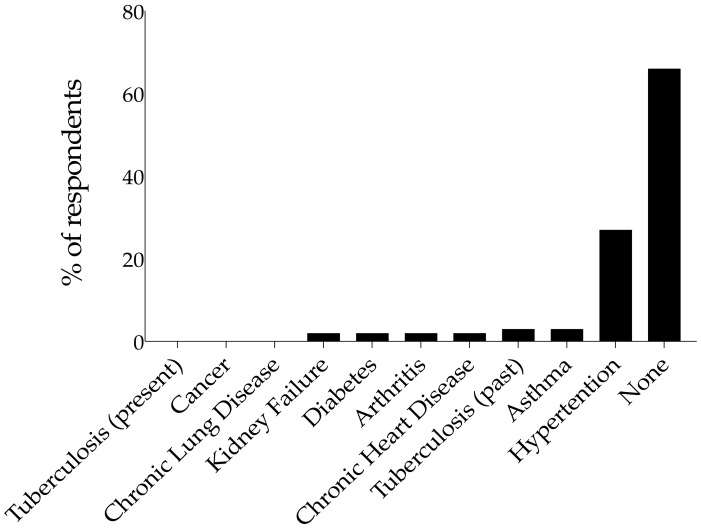
Prevalence of comorbidities reported (*n* = 62). Bars show the percentage of respondents that reported the respective comorbid diseases.

In response to the question “Do you have any of these diseases?” (Figure 3), 66% (*n* = 41) reported no underlying medical condition. Of those who reported a comorbid condition, a larger proportion (27%; *n* = 17) reported hypertension. There was no report of cancer, present tuberculosis, or chronic lung disease.

### 3.3. Clinical Manifestations and Management of Acute COVID-19 Infection

**Figure 4:**
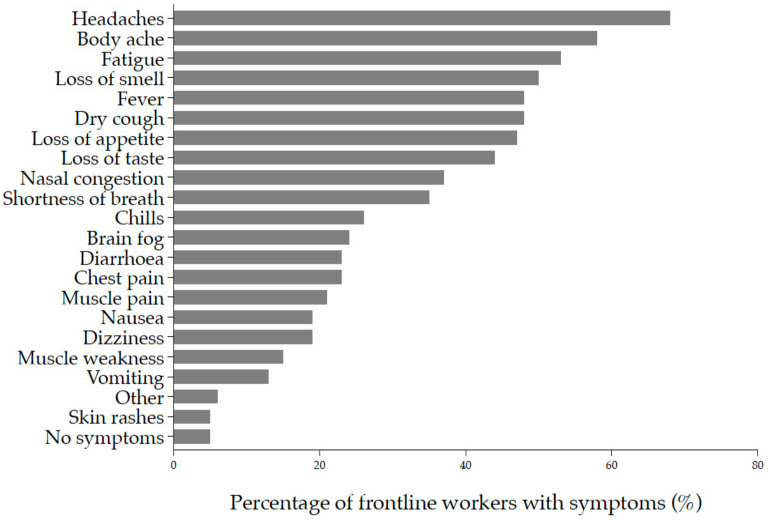
Acute COVID-19 symptoms were reported at the time of diagnosis (*n* = 62). Each symptom is ordered from top to bottom by increasing frequency of occurrence. The bars represent the proportion of workers with symptoms.

Symptoms reported at time of diagnosis included but were not limited to headache (68%; *n* = 42), body ache (58%; *n* = 36), fatigue (53%; *n* = 33), loss of smell (50%; *n* = 31), dry cough (48%; *n* = 30), fever (48%; *n* = 30), loss of appetite (47%; *n* = 29), and loss of taste (44%; *n* = 27) (Figure 4).

In terms of COVID-19 management after diagnosis, 55 (88.7%) self-isolated, 7 (11.3%) were admitted to hospital, 1 (1.6%) was admitted to a general ward for six days, and 3 (4.8%) were in an intensive care unit (ICU) for three, five, and six days each. For hospital admissions, none were on a ventilator, diagnosed with stroke, nor received dialysis for kidney failure, and one (1.6%) was sedated.

### 3.4. Clinical Symptoms Post-Acute COVID-19

**Figure 5:**
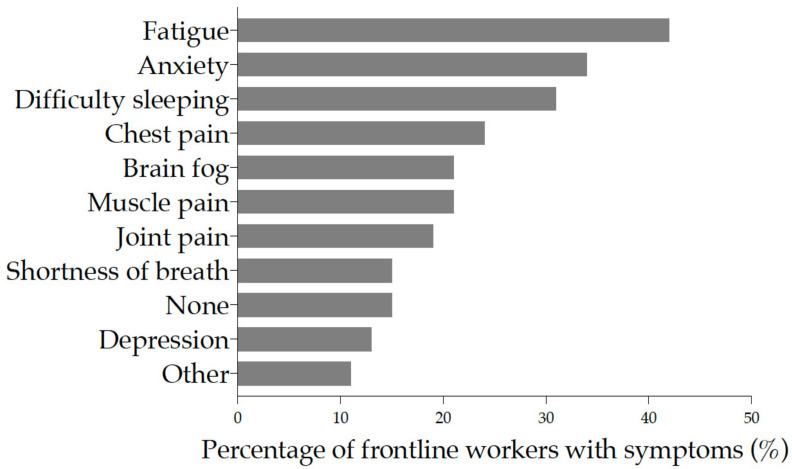
Reported post-acute COVID-19 symptoms among frontline workers. Each symptom is ordered from top to bottom by increasing frequency of occurrence. The bars represent the proportion of workers with symptoms.

Following post-acute COVID-19 infection, fatigue (42%), anxiety (34%), difficulty sleeping (31%), chest pain (24%), muscle pain, and brain fog (21% each) were the six major symptoms experienced by frontline workers. Joint pain was experienced by 19% of respondents (Figure 5) and was the most bothersome symptom (Supplementary Figure S1). In response to whether the symptoms had disappeared, the majority (46.8%) responded “No”. The duration of symptoms varied from one week to more than three months. Except for shortness of breath, all other symptoms were experienced for longer than a week (Supplementary Figure S2).

**Figure 6:**
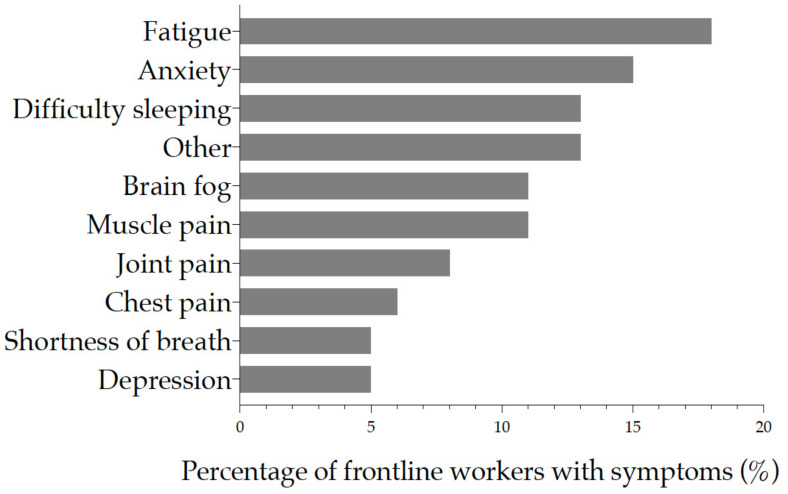
Post-acute COVID-19 symptoms were reported on the day of the survey (*n* = 62). Each symptom is ordered from top to bottom by increasing frequency of occurrence. The x-axis represents the proportion of workers with symptoms.

When asked which symptoms the health workers were currently experiencing and how severe on a scale of 0 (not noticeable) to 10 (unbearable) the symptoms were, 18% (*n* = 11) reported fatigue, 15% (*n* = 9) anxiety, and difficulty sleeping in 13% (*n* = 8) (Figure 6) with moderate severity.

The number of respondents who participated in some form of moderate to strenuous physical activity was 45 (73%) before COVID-19 diagnosis, and 38 (61%) post-acute COVID-19. Physical activity was performed at a mean of 3.4 (Standard Deviation [SD] = 1.7) days (95% CI: 2.6–4.0) before and 2.9 (SD = 1.5) days (95% CI: 2.3–3.4) after COVID-19 recovery. Similarly, physical activity was performed at a mean of 32.9 (SD = 22.8) minutes (95% CI: 25.0–40.9) before and 29.4 (SD = 19.0) minutes (95% CI: 22.8–36.0) post-acute COVID-19. However, there was no observed statistical difference between pre- and post-COVID-19 physical activity (*p* = 0.51; paired *t*-test).

Persistent (long) COVID-19 (symptoms experienced for three months and longer) was observed in 24.2% (15 out of 62) of the respondents. Of these, the median age was 40 years (IQR = 34–54 years) as opposed to 32 years (IQR = 29–43 years) for those without long COVID-19, 40% (*n* = 6) were aged 34–44 years, and 80% (*n* = 12) were female (Table 2). While there was an observed significant difference in age between frontline workers with long COVID-19 and those without, there were no statistical differences by sex, and ethnicity, and a near significant difference in smoking, alcohol, and recreational drug intake.

**Figure 7:**
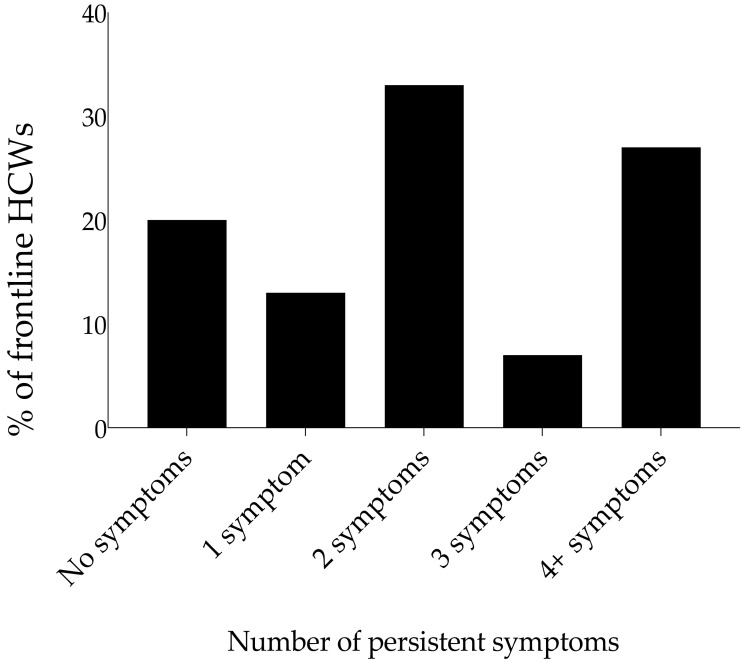
Persistent COVID-19 symptoms at three months and longer (*n* = 15). Number of symptoms remaining after 3 months post-acute infection for frontline workers experiencing persistent symptoms. Bars show the percentage of respondents that reported none or at least one symptom.

Of those who still experienced symptoms, 33% reported more than one persistent symptom (Figure 7). While there was no observed combination of symptoms that was prevalent for workers that experienced two symptoms and more, fatigue, anxiety, and muscle pain were common. To understand if any association existed between fatigue and all the confounding variables (age, sex, ethnicity, smoking, alcohol, and recreational drug intake), we observed no significant association with any of the factors (Supplementary Table S2).

## 4. Discussion

In this survey, we aimed to identify and determine the prevalence of persistent physical and/or mental health symptoms frontline workers at Right to Care (RTC) who have passed the acute phase of COVID-19 continue to experience. Our findings showed that more than a third of the respondents had symptoms for four weeks and beyond, and persistence of fatigue, anxiety, and difficulty sleeping with moderate severity (scale 2–5) was seen. Persistent symptoms post-acute COVID-19 are not uncommon. Similar findings have been reported elsewhere [12,13]. Compared to other studies [14,15], our sample population consisted mostly of non-hospitalised health care workers in the frontline of the pandemic.

We found that all frontline cadres had been affected by COVID-19 since its appearance and diffuse spread in South Africa. However, infection was more prevalent among community health workers and clinical personnel. This is concurrent with national COVID-19 statistics as well as elsewhere [16].

In our survey, 47% of respondents reported persistence in at least one symptom on the day of survey completion, particularly fatigue, anxiety, difficulty sleeping, muscle pain, and brain fog. These symptoms persisted for longer than three months in 24% of the frontline workers. Consistent with existing findings, fatigue was the most reported persistent and bothersome symptom post-acute SARS-CoV-2 infection [4,17,18].

We also found that there was no association of age, sex, ethnicity, alcohol, and recreational drug use with fatigue. In a post-acute COVID-19 Chinese study, the findings suggested sex differences, with women more likely to experience fatigue and anxiety/depression at 6 months of follow-up [4]. In the same study, age was positively associated with fatigue. Still in another study, the male gender was associated with a lower risk of post-COVID-19 syndrome [19], while the female gender was associated with long COVID-19 [20]. Regarding the level of physical activity, we found that for a minority of respondents, the level of physical activity reduced in frequency and duration, though not associated with long COVID-19. This could be a result of the lockdown regulations put in place in the first and second wave of the infection [21], not necessarily because of the presence of persistent symptoms.

This study was carried out among frontline workers at Right to Care, a multi-provincial organisation, which is likely to provide a more representative depiction of long COVID-19 within the South African context as opposed to the study [22,23] conducted solely in Cape Town City on patients with mild COVID-19 symptoms. The clinical presentation identified in this study can assist in creating more appropriate examination and management strategies in socio-economic conditions such as those found in the provinces covered in this study. Important considerations included in this study are the influence of smoking, alcohol, and drug intake on persistent symptoms. Additionally, this study clearly outlines the pre-morbid physical activity level and the change of this after acute COVID-19 infection.

There were a couple of limitations to this survey. Firstly, the retrospective nature of the study may have resulted in recall bias. Secondly, as the survey was distributed via different ways (email, WhatsApp, and SMS), restriction to recently diagnosed cases was inevitable, but that notwithstanding, the prevalence of long COVID-19 is reported for only workers who experienced symptoms for more than three months. Third, the small sample did not provide enough statistical power to determine risk factors for long COVID-19 in the study population. Lastly, we did not test the participants for an acute COVID-19 infection at the time of the survey, and the study did not indicate the management strategies used for the acute COVID-19 and Long COVID-19 phases. Despite these, the survey was able to answer the main survey objectives, but caution should apply when extrapolating the findings to the entire population with long COVID-19, especially because some of the symptoms reported may not be related to COVID-19 infection, but rather stress from the pandemic, especially for healthcare workers.

## 5. Conclusions

Our study has shown that some COVID-19 symptoms persist among frontline healthcare workers long after their initial acute phase of infection. Our population was mostly health workers who are in the frontline dealing with the pandemic above other existing infections such as TB and HIV. These health workers were mainly troubled with fatigue, sleep difficulties, and anxiety lasting more than three months; as such, the long-term effect of COVID-19 even among those that are beyond the active phase of infection could have a direct impact on healthcare practitioners’ quality of life and their ability to provide appropriate health service delivery. Given the rise in cases of COVID-19 in South Africa and the world at large, the prevalence of long COVID-19 is likely to be substantial in South Africa. This study highlights the importance of identifying individuals who present with long COVID-19. A greater body of research is required to create a more systematic approach to physical and mental health examinations to identify patients with long COVID-19. The appropriate management strategies for long COVID-19 are also poorly understood and require additional research. Due to the wide variety of clinical features identified in patients with long COVID-19, this research will require a multidisciplinary approach. Additionally, considerations that need to be investigated are the specific effects of medication, rehabilitation, vaccination, and other public health measures on patients with long COVID-19.

## Figures and Tables

**Table 1:** Characteristics of survey respondents, *n* = 62.

	*n*	%
Age (median {IQR})	33.5 (30–44)	
Sex		
Female	47	75.8
Male	15	24.2
Missing	0	0.0
Age Groups		
23–33	31	50.0
34–44	17	27.4
45–55	9	14.5
≥56	5	8.1
Missing	0	0.0
Ethnicity		
African	54	87.1
Caucasian	2	3.2
Coloured *	6	9.7
Missing	0	0.0
Smoking Status		
Current smoker	4	6.4
Never smoker	53	85.5
Past smoker	8	8.1
Missing	0	0.0
Alcohol Intake		
Daily	2	3.2
Occasional	24	38.7
Not at all	36	58.1
Missing	0	0.0
Recreational Drug Use		
Daily	1	1.6
Occasional	1	1.6
Not at all	60	96.8
Missing	0	0.0

* An official terminology in South Africa used to describe a multifaceted lived experience [11]. IQR, interquartile range; *n*, number; %, percentage.

**Table 2:** Characteristics of survey respondents with long COVID-19 (*n* = 15).

Demographics	N	Long COVID-19		*p*-Value
		No *n* (%), 47 (75.8)	Yes *n* (%), 15 (24.2)	
Median Age (IQR)	33.5 (30–44)	32 (29–43)	40 (34–54)	
Age Groups				***0.02***
23–33	31 (50.0)	28 (59.6)	3 (20.0)	
34–44	17 (27.4)	11 (23.4)	6 (40.0)	
45–55	9 (14.5)	6 (12.8)	3 (20.0)	
≥56	5 (8.1)	2 (4.2)	3 (20.0)	
Sex				*0.48*
Female	47 (75.8)	35 (74.5)	12 (80.0)	
Male	15 (24.2)	12 (25.5)	3 (20.0)	
Ethnicity				*0.47*
African	54 (87.1)	42 (892.4)	12 (80.0)	
Caucasian	2 (3.2)	1 (2.1)	1 (6.7)	
Coloured	6 (9.7)	4 (8.5)	2 (13.3)	
Smoking Status				*0.09*
Current smoker	4 (6.4)	4 (8.5)	0 (0.0)	
Never smoker	53 (85.5)	41 (87.2)	12 (80.0)	
Past smoker	5 (8.1)	2 (4.3)	3 (20.0)	
Alcohol Intake				*0.07*
Daily	2 (3.2)	0 (0.0)	2 (13.3)	
Occasional	24 (38.7)	20 (42.6)	4 (26.7)	
Not at all	36 (58.1)	27 (57.4)	9 (60.0)	
Recreational Drug Use				*0.06*
Daily	1 (1.6)	0 (0.0)	1 (6.7)	
Occasional	1 (1.6)	0 (0.0)	1 (6.6)	
Not at all	60 (96.8)	47 (100.0)	13 (86.7)	
Total	62	47	15	

IQR, interquartile range; N *n*, number; *p*-values italicised were obtained using Fisher’s exact test, the bold indicates statistical significance.

## Data Availability

The data presented in this study are available on request from the corresponding author. The data are not publicly available due to ethical reasons.

## Supplementary Materials

The following supporting information can be downloaded at: https://www.mdpi.com/article/10.3390/ijerph19105933/s1, Figure S1: Duration of post-acute COVID-19 symptoms among survey respondents; Figure S2: Reported bothersome/troubling post-acute COVID-19 symptoms among respondents; Table S1: Characteristics of RTC employees who tested for COVID-19; Table S2: Factors associated with fatigue for workers who experienced long COVID-19.

## References

[1] Dong E., Du H., Gardner L. (2020). An interactive web-based dashboard to track COVID-19 in real time. Lancet Infect. Dis..

[2] Ladds E., Rushforth A., Wieringa S., Taylor S., Rayner C., Husain L., Greenhalgh T. (2020). Persistent symptoms after COVID-19: Qualitative study of 114 “long Covid” patients and draft quality principles for services. BMC Health Serv. Res..

[3] Carfì A., Bernabei R., Landi F., Gemelli against COVID-19 Post-Acute Care Study Group (2020). Persistent Symptoms in Patients After Acute COVID-19. JAMA.

[4] Huang C., Huang L., Wang Y., Li X., Ren L., Gu X., Kang L., Guo L., Liu M., Zhou X. (2021). 6-month consequences of COVID-19 in patients discharged from hospital: A cohort study. Lancet.

[5] News24. https://www.news24.com/news24/SouthAfrica/News/coronavirus-511-health-workers-positive-26-hospitalised-and-2-have-died-zweli-mkhize-20200506.

[6] Han Q., Lin Q., Jin S., You L. (2020). Coronavirus 2019-nCoV: A brief perspective from the front line. J. Infect..

[7] WHO Fact Sheets on Physical Activity. https://www.who.int/news-room/fact-sheets/detail/physical-activity.

[8] Centres for Disease Control and Prevention Interim Guidelines for Collecting, Handling, and Testing Clinical Specimens from Persons for Coronavirus Disease 2019 (COVID-19). https://www.cdc.gov/coronavirus/2019-nCoV/lab/guidelines-clinical-specimens.html.

[9] Harris P.A., Taylor R., Thielke R., Payne J., Gonzalez N., Conde J.G. (2009). Research electronic data capture (REDCap)—A metadata-driven methodology and workflow process for providing translational research informatics support. J. Biomed. Inform..

[10] WHO. https://www.who.int/publications/i/item/WHO-2019-nCoV-Post_COVID-19_condition-Clinical_case_definition-2021.1.

[11] Nilsson 2016 Coloured by Race: A Study about Making Coloured Identities in South Africa. https://www.diva-portal.org/smash/get/diva2:939226/FULLTEXT01.pdf.

[12] Davis H.E., Assaf G.S., McCorkell L., Wei H., Low R.J., Re’em Y., Redfield S., Austin J.P., Akrami A. (2021). Characterizing long COVID in an international cohort: 7 months of symptoms and their impact. EClinicalMedicine.

[13] Ayoubkhani D., Khunti K., Nafilyan V., Maddox T., Humberstone B., Diamond I., Banerjee A. (2021). Post-COVID syndrome in individuals admitted to hospital with COVID-19: Retrospective cohort study. BMJ.

[14] Clift A.K., Coupland C., Keogh R.H., Diaz-Ordaz K., Williamson E., Harrison E.M., Hayward A., Hemingway H., Horby P., Mehta N. (2020). Living risk prediction algorithm (QCOVID) for risk of hospital admission and mortality from coronavirus 19 in adults: National derivation and validation cohort study. BMJ.

[15] Sani G., Janiri D., Di Nicola M., Janiri L., Ferretti S., Chieffo D. (2020). Mental health during and after the COVID-19 emergency in Italy. Psychiatry Clin. Neurosci..

[16] Garrigues E., Janvier P., Kherabi Y., Le Bot A., Hamon A., Gouze H., Doucet L., Berkani S., Oliosi E., Mallart E. (2020). Post-discharge persistent symptoms and health-related quality of life after hospitalization for COVID-19. J. Infect..

[17] Nalbandian A., Sehgal K., Gupta A., Madhavan M.V., McGroder C., Stevens J.S., Cook J.R., Nordvig A.S., Shalev D., Sehrawat T.S. (2021). Post-acute COVID-19 syndrome. Nat. Med..

[18] Di Stefano V., Battaglia G., Giustino V., Gagliardo A., D’Aleo M., Giannini O., Palma A., Brighina F. (2021). Significant reduction of physical activity in patients with neuromuscular disease during COVID-19 pandemic: The long-term consequences of quarantine. J. Neurol..

[19] Augustin M., Schommers P., Stecher M., Dewald F., Gieselmann L., Gruell H., Horn C., Vanshylla K., DiCristanziano V., Osebold L. (2021). Post-COVID Syndrome in Non-Hospitalised Patients with COVID-19: A Longitudinal Prospective Cohort Study. Lancet Reg. Health Eur..

[20] Sudre C.H., Murray B., Varsavsky T., Graham M.S., Penfold R.S., Bowyer R.C., Pujol J.C., Klaser K., Antonelli M., Canas L.S. (2021). Attributes and predictors of long COVID. Nat. Med..

[21] Shalash A., Roushdy T., Essam M., Fathy M., Dawood N.L., Abushady E.M., Elrassas H., Helmi A., Hamid E. (2020). Mental Health, Physical Activity, and Quality of Life in Parkinson’s Disease During COVID-19 Pandemic. Mov. Disord. Off. J. Mov. Disord. Soc..

[22] Mendelsohn A.S., De Sá A., Morden E., Botha B., Boulle A., Paleker M., Davies M.A. (2022). COVID-19 wave 4 in Western Cape Province, South Africa: Fewer hospitalisations, but new challenges for a depleted workforce. S. Afr. Med. J..

[23] Mendelsohn A.S., Nath N., De Sá A., Von Pressentin K.B. (2022). Two months follow-up of patients with non-critical COVID-19 in Cape Town, South Africa. S. Afr. Fam. Pract..

