# Supplementary material for: Persistent Symptoms among Frontline Health Workers Post-Acute COVID-19 Infection
Source: Int J Environ Res Public Health. 2022 May 13;19(10):5933. doi: 10.3390/ijerph19105933 (PMC9140668; doi:10.3390/ijerph19105933)
Supplement: Supplementary file 1 [file ijerph-19-05933-s001.zip › ijerph-1684434-supplementary.pdf]

Supplementary Table S1: Characteristics of RTC frontline health workers who tested for COVID-19

| Variables | N | COVID-19 Test Outcome | | *p*-Value |
|---|---|---|---|---|
| | | Positive n (%) | Other n (%) | |
| Staff Cadres | | | | *<0.01* |
| Community/Field health Workers | 329 | 95 (38.3) | 234 (35.0) | |
| Medical/Clinical personnel | 137 | 42 (16.9) | 95 (14.2) | |
| Data capturers/Clerks | 140 | 32 (13.0) | 108 (16.2) | |
| Programme Coordinators | 25 | 5 (2.0) | 20 (3.0) | |
| Programme Support Staff | 82 | 35 (14.1) | 47 (7.0) | |
| Missing | 203 | 39 (15.7) | 164 (24.6) | |
| Reasons for testing | | | | *0.01* |
| COVID-19 contact | 271 | 73 (29.4) | 198 (29.6) | |
| COVID-19 PUI/Suspect | 503 | 151 (60.9) | 352 (52.7) | |
| Routine Health Surveillance | 125 | 24 (9.7) | 101 (15.1) | |
| Travel requirements | 4 | 0 (0.0) | 4 (0.6) | |
| Missing | 13 | 0 (0.0) | 13 (2.0) | |
| Province | | | | *<0.01* |
| Gauteng | 62 | 16 (6.5) | 46 (6.9) | |
| Free State | 326 | 81 (33.0) | 245 (36.7) | |
| Mpumalanga | 249 | 61 (25.0) | 188 (28.1) | |
| Eastern Cape | 24 | 15 (6.1) | 9 (1.4) | |
| Northern Cape | 2 | 2 (1.0) | 0 (0.0) | |
| Western Cape | 248 | 70 (28.2) | 178 (26.7) | |
| North West | 5 | 3 (1.2) | 2 (0.3) | |
| Districts | | | | *<0.01* |
| City of Johannesburg MM | 33 | 8 (3.2) | 25 (4.0) | |
| City of Tshwane MM | 23 | 7 (2.8) | 16 (2.4) | |
| Lejweleputswa | 21 | 6 (2.4) | 15 (2.3) | |
| Ehlanzeni | 240 | 58 (23.4) | 182 (27.3) | |
| Gert Sibande | 11 | 2 (0.8) | 9 (1.4) | |
| Nelson Mandela Bay MM | 5 | 3 (1.2) | 2 (0.3) | |
| Amatole | 11 | 7 (3.0) | 4 (1.0) | |
| OR Tambo | 5 | 5 (2.0) | 0 (0.0) | |
| Thabo Mofutsanyane | 302 | 73 (29.4) | 229 (34.3) | |
| Dr Kenneth Kaunda | 7 | 6 (2.4) | 1 (0.2) | |
| Central Karoo | 104 | 15 (6.1) | 39 (6.0) | |
| Cape Winelands | 54 | 15 (6.1) | 39 (6.0) | |
| Overberg | 86 | 25 (10.0) | 61 (9.1) | |
| City of Cape Town MM | 2 | 0 (0.0) | 2 (0.3) | |
| Ngaka Modiri Molema | 1 | 1 (0.4) | 0 (0.0) | |
| Nkangala | 2 | 1 (0.4) | 1 (0.2) | |
| Missing | 9 | 1 (0.4) | 8 (1.2) | |
| **Total** | **916** | **248** | **668** | |

%, percentage; N n, number; *p* value obtained using Pearson Chi and Fisher's exact test

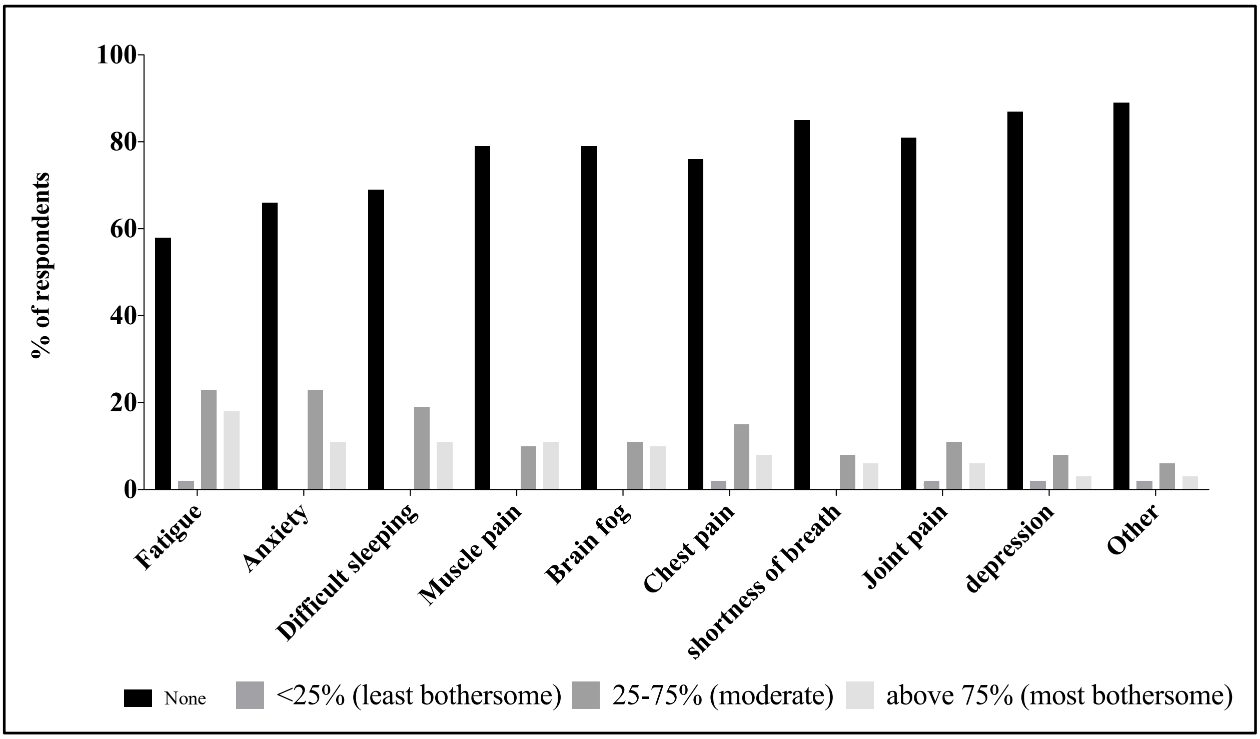


Supplementary Figure S1: Reported bothersome/troubling post-acute COVID-19 symptoms among respondents

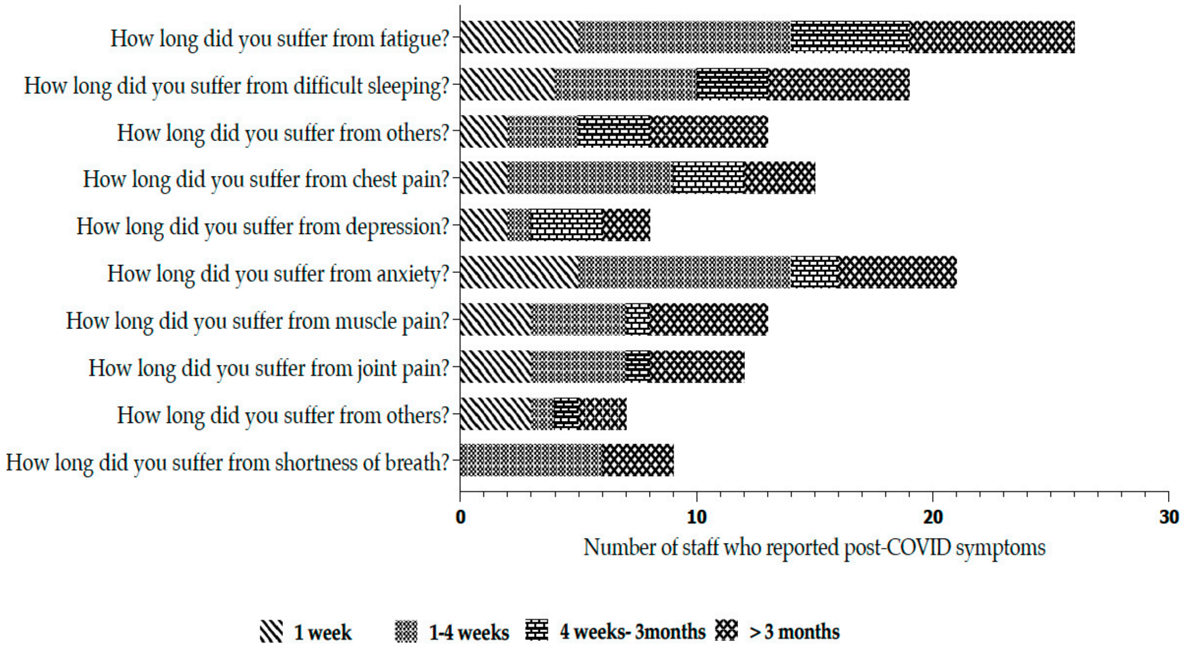


**Supplementary Figure S2: Duration of post-acute COVID-19 symptoms among survey respondents.** Stacked bar graphs represent duration of the different symptoms reported.

Supplementary Table S2 Factors associated with fatigue for workers who experienced long COVID.

| Variables | N | Fatigue | | *p*-Value |
|---|---|---|---|---|
| | | No<br>n (%), 9 (60) | Yes<br>n (%), 6 (40) | |
| Age Groups | | | | ***0.94*** |
| 23–33 | 3 (20.0) | 2 (22.2) | 1 (16.7) | |
| 34–44 | 6 (40.0) | 3 (33.3) | 3 (16.7) | |
| 45–55 | 3 (20.0) | 2 (22.2) | 1 (16.7) | |
| ≥56 | 3 (20.0) | 2 (22.2) | 1 (16.7) | |
| **Sex** | | | | *0.29* |
| Female | 12 (80.0) | 8 (88.9) | 4 (66.7) | |
| Male | 3 (20.0) | 1 (11.1) | 2(33.3) | |
| **Ethnicity** | | | | *0.29* |
| African | 12 (80.0) | 6 (66.7) | 6(100.0) | |
| Caucasian | 1 (6.7) | 1 (11.1) | 0 (0) | |
| Coloured* | 2 (13.3) | 2 (22.2) | 0(0) | |
| **Smoking Status** | | | | 0.79 |
| Current smoker | 0 (0.0) | 0 (0.0) | 0 (0.0) | |
| Never smoker | 12 (80.0) | 7 (77.8) | 12 (80.0) | |
| Past smoker | 3(20.0) | 2 (22.2) | 3 (20.0) | |
| **Alcohol Intake** | | | | *0.46* |
| Daily | 2 (13.3) | 2 (22.2) | 0 (0) | |
| Occasional | 4 (26.7) | 2 (22.2) | 2 (33.3) | |
| Not at all | 9 (60) | 5 (55.6) | 4 (66.7) | |
| **Recreational Drug Use** | | | | *0.46* |
| Daily | 1 (6.7) | 1(11.1) | 0 (0) | |
| Occasional | 13 (86.7) | 7 (77.8) | 6 (100) | |
| Not at all | 1 (6.7) | 1 (11.1) | 0 (0) | |

**an official terminology in South Africa used to describe a multifaceted lived experience (Nilson, 2016)*. IQR, interquartile range; N n, number; *p* value obtained using Fisher's exact test
